# Imaging characteristics of pediatric primary thymic lymphoepithelioma-like carcinoma: case reports of four children with a literature review

**DOI:** 10.3389/fped.2025.1494946

**Published:** 2025-02-21

**Authors:** Xiao-dong Zhu, Yuan Fang, Xiao-yu Wang, Jun Hu, Dong-hao Gu, Qiong Yao, Zhong-wei Qiao

**Affiliations:** ^1^ Department of Radiology, Anhui Provincial Children’s Hospital, Hefei, China; ^2^ Department of Radiology, Children’s Hospital of Fudan University, Shanghai, China

**Keywords:** children, lymphoepithelioma-like carcinoma, computed tomography, case report, tumor

## Abstract

**Objective:**

This study aimed to analyze the imaging characteristics of lymphoepithelioma-like carcinoma (LELC) in children's thymi.

**Methods:**

Four pediatric cases of primary thymic LELC confirmed by needle biopsy pathology from our research center were enrolled. All children underwent an enhanced chest computed tomography (CT) examination. In addition, 11 cases of pediatric thymic LELC with CT manifestations reported in the literature over the past 20 years were combined to explore their imaging characteristics.

**Results:**

In our research center, there were four cases, all boys, with an average age of 11.25 ± 2.87 years. CT showed a soft tissue mass in the anterior mediastinum in all four cases, with the mass being lobulated or roughly circular and growing laterally. The largest mass had a diameter of 120 mm, with an average of 90 ± 21.6 mm. In three cases, the mass showed cystic necrosis, which enhanced unevenly, and in three cases, small blood vessels were seen traversing the mass. Two cases had an invasion of the pleura and lung with associated pleural effusion. In two cases, vascular reconstruction showed blood supply from branches of the internal thoracic artery, with one case having spinal metastasis. Combined with literature reports of 11 pediatric cases with thymic LELC, a total of 15 cases of thymic LELC were identified: 13 cases were boys, 2 cases were girls, and the average age was 11.2 ± 2.9 years. The largest lesion had a diameter of 160 mm, with an average of 107 ± 27.78 mm. Seven cases had cystic necrosis, 4 cases had calcification, 5 cases did not mention the enhancement method, and the remaining 10 cases showed uneven enhancement. Furthermore, six cases had tumor invasion of adjacent large blood vessels; six cases had pleural effusion; five cases had pleural invasion; six cases had metastasis to the mediastinal, hilar, or axillary lymph nodes cases had pulmonary metastasis; and four cases had bone metastasis.

**Conclusion:**

Thymic LELC in children showed a higher incidence in boys. The imaging characteristics of pediatric thymic LELC manifested as a large mass located in the anterior mediastinum, with highly malignant features and metastasis.

## 1 Introduction

Thymic carcinoma can be divided into 12 subtypes, among which lymphoepithelioma-like carcinoma (LELC) is one, and its pathological tissue is similar to that of undifferentiated nasopharyngeal carcinoma (1). LELC has been reported in various organs, including the lungs, skin, and parotid glands, but it is very rare in the thymus, with only case reports available. We have collected a total of four cases of pediatric thymic LELC in our medical center and reviewed their clinical symptoms, imaging characteristics, treatment, and prognosis. We also conducted a retrospective analysis of 11 cases of pediatric thymic LELC with computed tomography (CT) findings or image reports in the literature over the past 20 years to analyze the imaging characteristics.

## 2 Materials and methods

All four patients were enrolled in our research center from 2019 to 2024, with complete clinical, pathological, and imaging data. The follow-up date cut-off was 10 July 2024.

For the CT examinations, scanning was performed using a Philips Brilliance 64-slice CT, with iodixanol (270 mg/ml). The injection dosage was 2–3 ml/kg and administered via antecubital vein injection. The tube voltage was 100 kV, tube current 60 mAs, rotation speed 0.8 s/rot, pitch 0.992:1, and slice thickness 0.625 mm.

For the MRI examinations, a 1.5 T MRI scanner (Philips Medical Systems, Netherlands) was used, with examination sequences including transverse T1-weighted images (T1WIs, TR 400 ms, TE 8 ms), T2-weighted images (T2WIs, TR 2000 ms, TE 97 ms), T2WIs with fat suppression, and coronal and sagittal T1WIs and T2WIs.

The literature search strategy is shown in Table 1. In this review, we searched the PubMed database and the China National Knowledge Infrastructure (CNKI) database for results up to July 2024. The search keywords included “lymphoepithelioma-like thymic carcinoma” and “thymic lymphoepithelial carcinoma.”

**Table 1:** Literature search strategy.

Item	Content
Time	From January 2006 to July 2024
Databases	PubMed database and the China National Knowledge Infrastructure (CNKI) database
Keywords	“lymphoepithelioma-like thymic carcinoma” and “thymic lymphoepithelial carcinoma”
Inclusion criteria	We selected cases with detailed imaging findings and excluded cases with incomplete imaging descriptions or no imaging pictures from our research center.

## 3 Results

### 3.1 Clinical data

The four patients in this study ranged from 7 to 13 years old, with an average age of 11.25 ± 2.87 years old. All four cases were boys. The first two cases presented with non-specific symptoms such as cough and chest distress; both underwent chemotherapy, with one also receiving radiotherapy. The first case was lost to follow-up after 19 months, while the second case survived for 42 months. The third case was admitted with acute appendicitis; a preoperative chest x-ray suggested a mediastinal mass. Laparoscopic appendectomy was performed, followed by right thoracotomy and resection of the right mediastinal tumor. Postoperative positron emission tomography-CT (PET-CT) from another hospital showed residual lesions, and a second surgery was performed. The tumor has not recurred after 13 months of follow-up. The fourth case was admitted with a 3-month history of coughing up blood-tinged sputum, weight loss, and chest pain. CT suggested a mediastinal mass with hilar lymph nodes and bilateral lung metastasis, and chemotherapy was administered. The child is currently doing well after 6 months of follow-up. In addition, a review of the literature from the past 20 years reported a total of 11 cases of pediatric thymic LELC (2–8), with 9 boys and 2 girls; the average age was 11.18 ± 3.09 years (Table 2).

### 3.2 Imaging results

**Figure 1:**
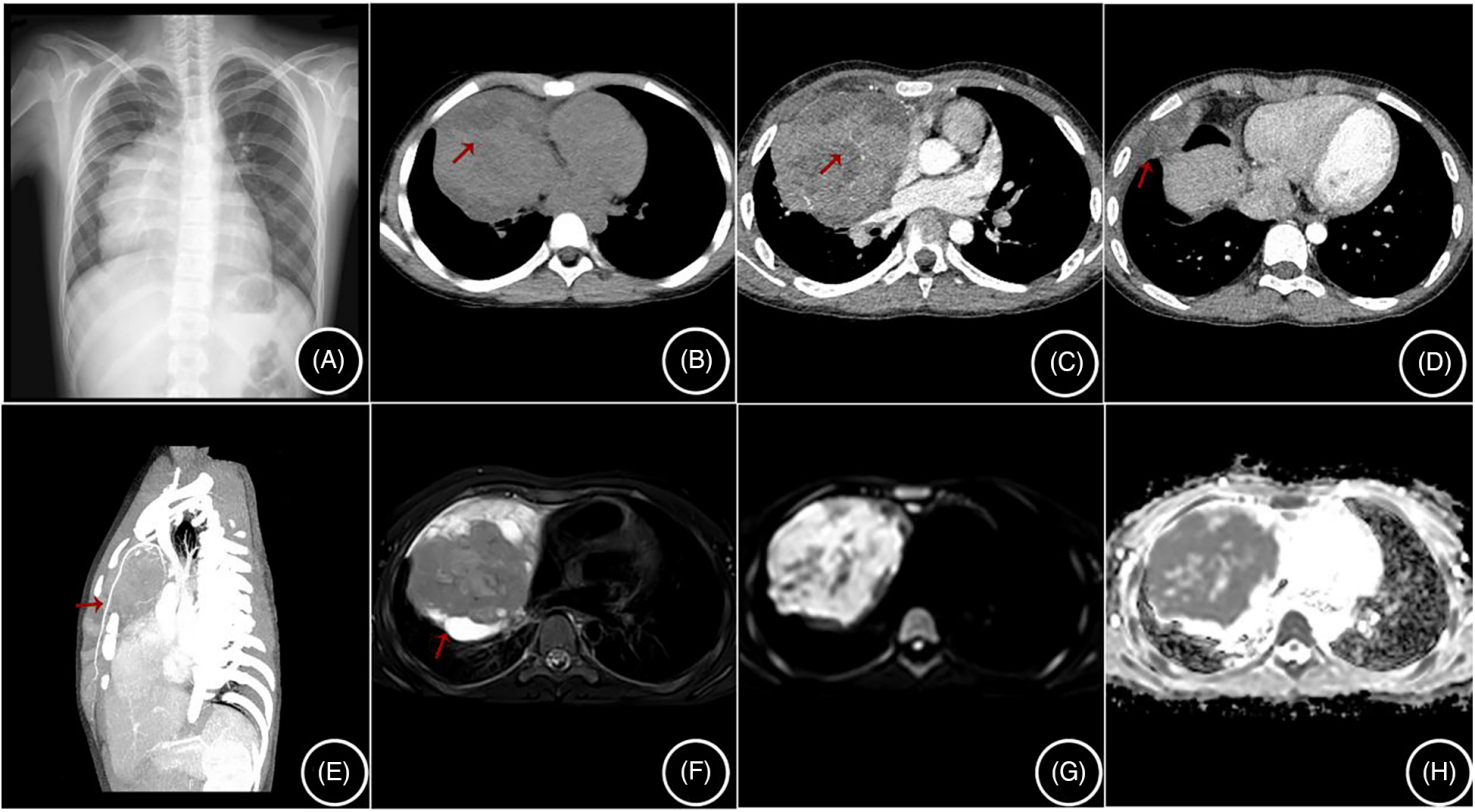
**(A–H)** are from the same case: **(A)** shows a mediastinal mass on a chest radiograph; **(B)** shows cystic necrosis (arrow) on a non-contrast CT scan; **(C)** shows tortuous small vessels within the enhanced lesion (arrow); **(D)** shows invasion of the right pleura (arrow); **(E)** shows the lesion being supplied by branches of the internal thoracic artery; **(F)** shows mixed signals in the lesion on MRI T2 fat-saturated images, with cysts showing high signal; and **(G,H)** show high signal in the solid components of the lesion on DWI and low signal on ADC.

**Figure 2:**
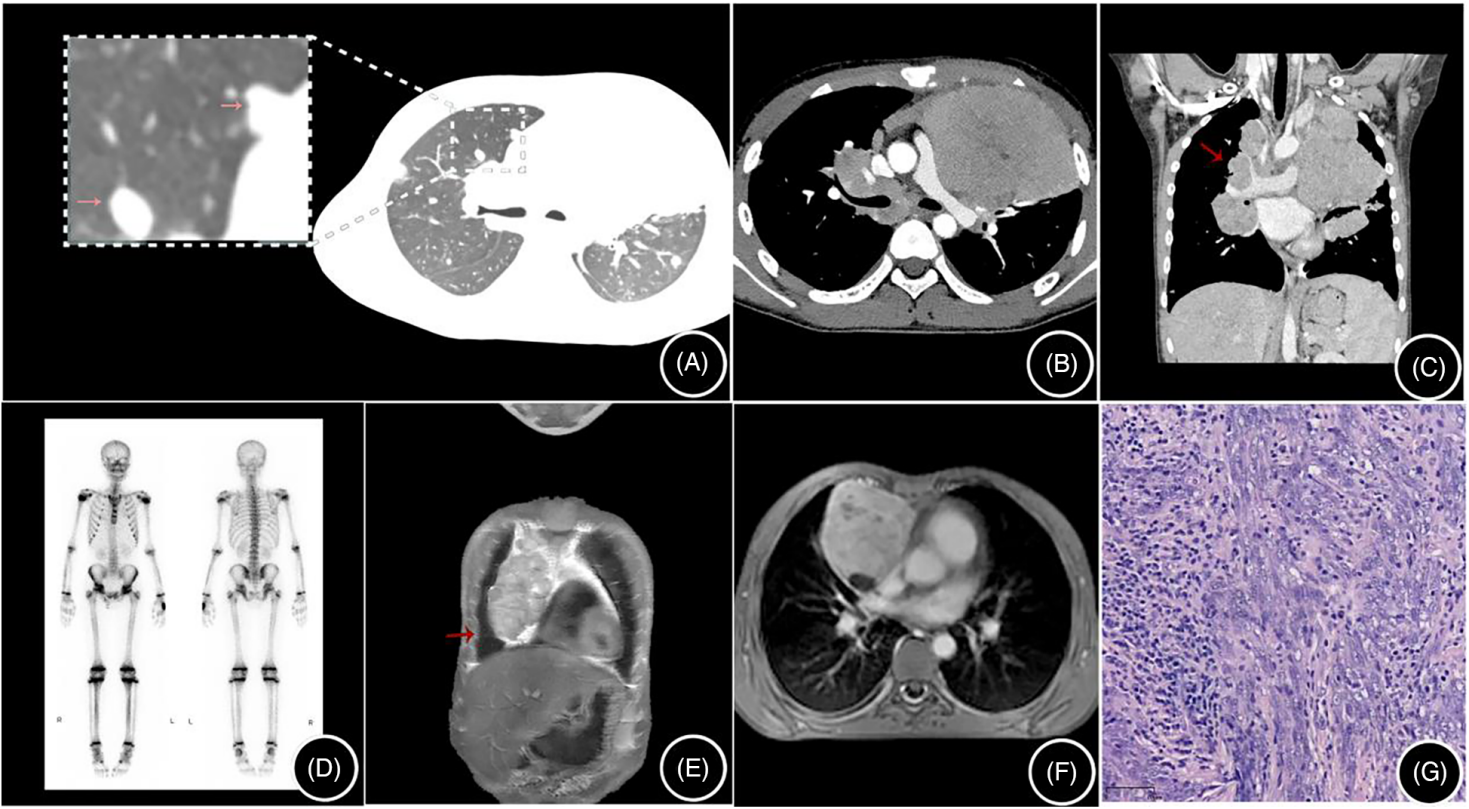
**(A–D)** are from the same case: **(A)** shows multiple pulmonary metastatic lesions (arrows) on a non-contrast CT scan; **(B)** is an enhanced CT scan showing a mediastinal mass; **(C)** shows multiple enlarged mediastinal and hilar lymph nodes (arrows); and **(D)** is an SPECT showing no bone metastasis. **(E,F)** are from the same case: **(E)** is a T2-weighted fat-saturated image with a high signal in the cysts; **(F)** is a contrast-enhanced MRI image showing heterogeneous enhancement of the lesion; and **(G)** shows tumor cells arranged in bundles and trabeculae, with a lymphocyte-rich stroma, H&E × 100.

All four cases showed a soft tissue mass in the anterior mediastinum on CT scans, with the mass being lobulated or roughly circular and growing toward one side. The largest lesion measured 120 mm in diameter, with an average of 90 ± 21.6 mm. The lesion density was heterogeneous, with three cases showing cysts and necrotic areas (Figure 1A). After contrast enhancement, the lesions showed inhomogeneous enhancement, with three cases showing vessels traversing within the tumor (Figure 1C), three cases showing encircling blood vessels (Figure 2C), two cases with the internal thoracic artery branches supplying the tumor (Figure 1E), two cases of pleural invasion (Figure 1D) and lung invasion (Figure 2A) accompanied by pleural effusion, and one case with spinal metastasis. Two cases underwent MRI examination, showing low T1WI signals and slightly high T2WI signals, with the solid part of the tumor showing mixed signals on the T2WI fat suppression sequence, and the cysts showing high signal, resembling a “grape cluster” sign (Figure 1F). After enhancement, the lesions showed inhomogeneous enhancement (Figure 2F), with the tumor exhibiting diffusion restriction: a high signal on diffusion-weighted imaging (DWI), and a low signal on apparent diffusion coefficient (ADC) (Figures 1G,H). Considering the high probability of bone metastasis in thymic carcinoma, we performed a single photon emission computed tomography (SPECT) examination on one case (Figure 2D).

### 3.3 Literature research and analysis of 15 cases

A total of three Chinese articles (7 cases) and four English articles (4 cases) were identified, along with 4 cases from our research center, totaling 15 cases of thymic LELC (Tables 2, 3). Among them, 13 cases were boys and 2 were girls, with an average age of 11.2 ± 2.9 years; all 15 cases of thymic LELC were located in the anterior mediastinum, with the largest diameter of the lesion being 160 mm and an average of 107 ± 27.78 mm. Furthermore, seven cases had cystic necrosis, four cases had calcification, and six cases had pleural effusion. In the enhanced scans, 4 cases did not mention the enhancement method, while the remaining 10 cases showed heterogeneous enhancement. Six cases had tumor invasion of adjacent large vessels, six cases had pleural effusion, five cases had pleural invasion, and six cases had metastasis to the mediastinal, hilar, or axillary lymph nodes. Five cases had pulmonary metastasis and four cases had bone metastasis. Epstein–Barr (EB) virus testing was conducted for the 15 pediatric patients, including tumor EB virus-encoded RNA, viral DNA detection, or antibodies, and all cases were related to the EB virus.

**Table 2:** Data from our research center.

	Research center cases (four cases)			
	1	2	3	4
Age (year old)/sex	7/male	13/male	12/male	13/male
Clinical manifestations	Cough, chest tightness	Cough, chest tightness	Acute appendicitis	Cough with hemoptysis, weight loss
Lesion location	Right anterior mediastinum	Right anterior mediastinum	Right anterior mediastinum	Left anterior mediastinum
Size (cm^3^)	8 × 9 × 11	7 × 9 × 10	9 × 8 × 10	12 × 8 × 9
Lesion Morphology	Round	Round	Round	Lobulated
Calcification	−	−	−	−
Cystic degeneration and necrosis	+	−	+	+
Enhancement pattern	Inhomogeneous	Inhomogeneous	Inhomogeneous	Inhomogeneous
Pleural and pericardial effusion	−	−	Bilateral pleural effusion	Bilateral pleural effusion
Lymph node metastasis	−	−	−	Mediastinal and hilar lymph nodes
Pulmonary metastasis	−	−	Bilateral lungs	Bilateral lungs
Bone metastasis	−	−	Spinal metastasis	−
Stage	Ⅲ	Ⅱ	Ⅳb	Ⅳb
Treatment	Chemotherapy	Chemotherapy + radiotherapy	Surgical resection + chemotherapy	Chemotherapy
Follow-up outcome	19 months, alive, lost to follow-up	42 months, alive	13 months, alive	6 months, alive

**Table 3:** Data from cited literature.

	Yaris et al. (2006) (2)	Tacyildiz et al. (2007) (3)	Kiliś-Pstrusińska et al. (2008) (4)	Liang et al. (2016) (5)	Pan et al. (2019) (6)	Congyong et al. (2022) (7)	Lixin et al. (2024) (8)				
							1	2	3	4	5
Age (years old)/sex	16/female	10/male	16/male	12/male	7/male	10/male	12/male	6/male	12/male	11/female	11/male
Clinical manifestations	Chest pain, cough, difficulty breathing	Chest pain, difficulty breathing	Cough	Chest tightness	Cough	Lower limb weakness, fever	Fever	Chest pain	Chest pain, cough, difficulty breathing	Chest pain, difficulty breathing	Cough
Lesion location	Right anterior mediastinum	Left anterior mediastinum	Anterior mediastinum	Right anterior mediastinum	Right anterior mediastinum	Left anterior mediastinum	Anterior mediastinum	Anterior mediastinum	Anterior mediastinum	Anterior mediastinum	Anterior mediastinum
Size (cm^3^)	12 × 12 × 10	10 × 10 × 15	N	12 × 12 × 10	16 × 10 × 12	9 × 5 × 6	12 × 6 × 9	13 × 8 × 10	15 × 12 × 16	8 × 6 × 12	8 × 8 × 11
Lesion morphology	Round	Round	Round	Round	Round	Lobulated	Lobulated	Lobulated	Lobulated	Lobulated	Lobulated
Calcification	N	N	N	punctate calcification	N	N	Little punctate calcification	Little punctate calcification	Little punctate calcification	−	−
Cystic degeneration and necrosis	+	N	N	N	N	N	+	−	+	−	+
Enhancement pattern	Inhomogeneous	N	N	N	N	N	Inhomogeneous	Inhomogeneous	Inhomogeneous	Inhomogeneous	Inhomogeneous
Pleural and pericardial effusion	Left pleural effusion	−	Bilateral pleural effusion	Right pleural metastasis	Right diaphragmatic dome and pleural metastasis	−	−	Bilateral pleural effusion	Right pleural effusion and metastasis	−	Left pleural effusion
Lymph node metastasis	Multiple enlarged lymph nodes in the upper mediastinum	−	−	−	−	Lymph node	−	Axillary lymph node metastasis	Supraclavicular lymph node	Lymph node	−
Pulmonary metastasis	Left lower lobe	−	−	Right lung	−	Left upper lobe	−	−	−	−	−
Bone metastasis	Sternum	−	−	−	−	−	−	−	−	Multiple Bone Metastases	Multiple Bone Metastases
Stage	IVb	III	IVa	IVa	IVa	III	III	IVb	IVb	IVb	IVb
Treatment	Chemotherapy + radiotherapy	Resection + radiotherapy + chemotherapy	Chemotherapy	Resection + chemotherapy + radiotherapy	Radiotherapy	Resection + chemotherapy	Chemotherapy	Chemotherapy	Chemotherapy	Chemotherapy	Chemotherapy
Follow-up outcome	15 months, dead	12 months, alive	11 months, dead	7 months, dead	N	29 months, alive	20 months, alive	12 months, dead	11 months, dead	1 month, alive	3 months, alive

N, not found.

## 4 Discussion

Thymic LELC is a rare primary tumor of the thymus (9). It is an aggressive tumor with a poorer prognosis compared to other mediastinal tumors. This rare epithelial tumor has been reported in various organs, such as the skin, lacrimal and salivary glands, thyroid, and vagina (10).

Three cases in our center were misdiagnosed as lymphoma or pleuropulmonary blastoma, and the possibility of lymphoma or thymic carcinoma was suspected in one case. Considering that thymic carcinoma has a high rate of bone metastasis up to 29% (2), we performed SPECT bone scans on the children. All four cases of thymic LELC in our center were located in the anterior mediastinum. CT scans showed cysts and necrosis, with inhomogeneous enhancement after contrast, and small vessels traversing within the tumor. MRI showed low T1 and slightly high T2 signals, with the cysts in the tumor showing a high signal on T2WI fat suppression sequences, resembling a “grape cluster” sign and inhomogeneous enhancement of the lesion after contrast. The solid components of the tumor were restricted in diffusion, with a high signal on DWI and a low signal on ADC, consistent with previous reports (11). Due to its high malignancy, thymic LELC often wraps around mediastinal vessels and grows invasively, directly invading adjacent tissues and organs, such as the pleura, lungs, diaphragm, and pericardium (11). We had three cases with growths encircling the surrounding blood vessels, two cases with invasion of the pleura and lungs accompanied by pleural effusion, and one case with spinal metastasis.

Currently, there are few radiological reports on pediatric thymic LELC. This study analyzes the radiological manifestations of thymic LELC in children, combined with a literature review, and summarizes the following characteristics. (1) Pediatric thymic LELC presents as a mixed cystic-solid mass located in the anterior mediastinum. (2) When the tumor is large, it often shows lobulated margins. In this research, the largest lesion had a diameter of 160 mm, with an average of 107 ± 27.78 mm, resembling adult thymic carcinoma. (3) Some of the pediatric thymic LELC masses had spotty calcifications at the edge (4/15), similar to adult thymic carcinoma (12), which can be distinguished from the arcuate and eggshell calcifications of thymoma, with tortuous small vessels inside the tumor. (4) The solid part of the tumor has a similar CT value to that of the thymus, and MRI shows low T1 and slightly high T2 signals, with light to moderate heterogeneous enhancement on CT and MRI. (5) The tumor usually has an abundant blood supply but is often combined with cystic necrosis. In this study, thick tortuous branches from the internal thoracic artery were observed in two cases. If there is a feeding artery inside the tumor, necrosis is often not common. The necrosis in thymic LELC in this group may be related to the tumor's large volume or rapid growth. (6) The tumor often grows around adjacent large vessels, easily invading vessels and the pleura. CT and MRI examinations observed that in this study six cases had invasion of adjacent large vessels, five cases had pleural invasion, and six cases had these combined with pleural effusion, slightly higher than that of adult thymic carcinoma (12). (7) The tumor often has local infiltration of adjacent structures or distant metastasis (14/15). SPECT and PET-CT can also observe whether the tumor has distant metastasis, which is important for clinical staging and treatment. Moreover, due to the high malignancy of thymic LELC, metastasis can also occur during chemotherapy, so SPECT and PET-CT play an important role in diagnosis and treatment (13, 14).

Differential diagnoses mainly include the following. (1) Lymphoma, especially with imaging manifestations similar to the high-incidence T-lymphoblastic lymphoma in children, is a differential diagnosis. However, T-lymphoblastic lymphoma often presents as diffuse uniform nodular or mass-like lesions, and calcification and necrosis are uncommon (15). Lymphoma grows around the pericardium and large blood vessels but is mostly centered around the aortic arch and tends to grow laterally whereas thymic LELC is positioned higher than lymphoma, grows laterally, and often presents as lobulated. Lymphoma shows severe compression and displacement of large blood vessels and the heart whereas thymic LELC often grows around large blood vessels and the heart, with a lesser degree of compression than lymphoma. Both mediastinal lymphoma and thymic LELC can involve the pericardium and pleura, but this is more common in thymic LELC. (2) Thymoma, which usually presents as a smooth or lobulated mass involving one side of the thymus, but may also involve both sides, is another differential diagnosis. Thymomas often show uniform enhancement and only a few, when associated with hemorrhage, necrosis, cystic areas, or calcification, show heterogeneous enhancement. Due to their lower malignancy, thymomas are less likely to involve vascular invasion and pleural or pericardial involvement (14). The main difference between thymoma and thymic carcinoma is based on histological grounds, leading to thymic carcinoma showing malignant features and different genetic immunohistochemical characteristics (16). (3) Neurogenic tumors, which may show cystic necrosis, calcification with pleural effusion formation on non-enhanced scans, and heterogeneous enhancement, are a third differential diagnosis. Among them, those with high malignancy may develop multiple metastases early, with bone metastasis being the most common, followed by lymph nodes, liver, etc. However, neurogenic tumors in the mediastinum are often spindle-shaped solid masses along the paraspinal sympathetic nerve chain in the posterior mediastinum (15), which is different from thymic LELC.

Thymic LELC is a rare and highly aggressive tumor that is seldom seen and is prone to misdiagnosis. Clinical characteristics show a predominant prevalence in adolescent boys aged 7–16 years old, large tumor size, high incidence of metastasis, late clinical staging, and poor clinical prognosis. CT scans of thymic LELC have characteristic manifestations and can accurately display the invasion of the lesions and adjacent structures, guiding clinical staging and treatment.

## Data Availability

The original contributions presented in the study are included in the article/Supplementary Material, further inquiries can be directed to the corresponding authors.

## References

[1] Kawagishi S Ose N Minami M Funaki S Kanou T Kimura K Total thymectomy for thymic lymphoepithelioma-like carcinoma—report of two cases. Surg Case Rep. (2019) 5:1–6. 10.1186/s40792-019-0706-6 31655916 PMC6815289

[2] Yaris N Nas Y Cobanoglu U Yavuz MN. Thymic carcinoma in children. Pediatr Blood Cancer. (2006) 47:224–7. 10.1002/pbc.20468 16007580

[3] Tacyildiz N Ugur H Yavuz G Unal E Comba A Okten İ The coexistence of thymic carcinoma and multiple granulomas in a Turkish child. Pediatr Hemat Oncol. (2007) 24:301–7. 10.1080/08880010701440882 17613873

[4] Kiliś-Pstrusińska K Medyńska A Zwolińska D Dobaczewski G. Lymphoepithelioma-like thymic carcinoma in a 16-year-old boy with nephrotic syndrome—a case report. Pediatr Nephrol. (2008) 23:1001–3. 10.1007/s00467-007-0666-0 18046582

[5] Liang S Wentian Z Na Y Gaoyan W. Thymic lymphoepitheliomalike carcinoma in children: one case report and literature review. J China Pediatr Blood Cancer. (2016) 21:305–9. 10.3969/j.issn.1673-5323.2016.06.006

[6] Pan H Song L Sun LR. Nephrotic syndrome: first presentation of lymphoepithelioma-like thymic carcinoma. Br J Hosp Med. (2019) 80:52–3. 10.12968/hmed.2019.80.1.52 30592665

[7] Congyong L Yuqiang L Yanqi Y. Thymic lymphoepithelioma-like carcinoma in a child: one case report. Chin J Thorac Cardiovasc Surg. (2022) 38:619–21. 10.3760/cma.j.cn112434-202110624-00213

[8] Lixin Y Di HU Zhonglong H Shuangfeng Y Yun P. CT Manifestations and clinical features of thymic lymphoepithelioma-like carcinoma in children and literature review. J Pract Radiol. (2024) 40:789–92. 10.3969/j.issn.1002-1671.2024.05.025

[9] Kedilaya S Revanth RB Balije S. Thymic lymphoepithelial carcinoma: a rare aggressive mediastinal mass. Cureus J Med Sci. (2024) 16(7):e64337. 10.7759/cureus.64337 PMC11316522 39130854

[10] Liu Z Feng Y Xiao Y Zhang X Li J Xie F Clinical characteristics, prognostic factors, and treatment modalities for head and neck lymphoepithelioma-like carcinoma: a real-world study from southern China. Radiother Oncol. (2023) 187:109814. 10.1016/j.radonc.2023.109814 37480992

[11] Ose N Kawagishi S Funaki S Kanou T Fukui E Kimura K Thymic lymphoepithelial carcinoma associated with Epstein-Barr virus: experiences and literature review. Cancers. (2021) 13:4794. 10.3390/cancers13194794 34638279 PMC8507618

[12] Mayoral M Pagano AM Araujo-Filho JAB Zheng J Perez-Johnston R Tan KS Conventional and radiomic features to predict pathology in the preoperative assessment of anterior mediastinal masses. Lung Cancer. (2023) 178:206–12. 10.1016/j.lungcan.2023.02.014 36871345 PMC10544811

[13] Akamine T Nakagawa K Ito K Watanabe H Yotsukura M Yoshida Y Impact of 18F-FDG PET on TNM staging and prognosis in thymic epithelial tumors. Ann Surg Oncol. (2024) 31:192–200. 10.1245/s10434-023-14328-z 37743455

[14] Strange CD Ahuja J Shroff GS Truong MT Marom EM. Imaging evaluation of thymoma and thymic carcinoma. Front Oncol. (2022) 11:810419. 10.3389/fonc.2021.810419 35047412 PMC8762255

[15] Ozawa Y Hiroshima M Maki H Hara M Shibamoto Y. Imaging findings of lesions in the middle and posterior mediastinum. Jpn J Radiol. (2021) 39:15–31. 10.1007/s11604-020-01025-0 32740793

[16] Cabezón-Gutiérrez L Pacheco-Barcia V Carrasco-Valero F Palka-Kotlowska M Custodio-Cabello S Khosravi-Shahi P. Update on thymic epithelial tumors: a narrative review. Mediastinum. (2024) 8:33–33. 10.21037/med-23-47 38881809 PMC11176988

