# Supplementary material for: Imaging characteristics of pediatric primary thymic lymphoepithelioma-like carcinoma: case reports of four children with a literature review
Source: Front Pediatr. 2025 Feb 21;13:1494946. doi: 10.3389/fped.2025.1494946 (PMC11885277; doi:10.3389/fped.2025.1494946)
Supplement: Supplementary file 1 [file Datasheet1.pdf]

	Reference [2], 2006	Reference [3], 2007	Reference [4], 2008	Reference [5], 2016	Reference [6], 2022	Reference [7]					Research Center Cases (4 cases)			
						1	2	3	4	5	1	2	3	4
Age (year old)/Gender	16 /Female	10 /Male	16 /Male	12 /Male	10 /Male	12 /Male	6 /Male	16 /Female	10 /Male	16 /Male	7 /Male	13 /Male	12 /Male	13 /Male
Clinical Manifestations	Chest pain, cough, difficulty breathing	Chest pain, difficulty breathing	Cough	Chest tightness	Lower limb weakness, fever	Fever	Chest pain	Chest pain, cough, difficulty breathing	Chest pain, difficulty breathing	Cough	Cough, chest tightness	Cough, chest tightness	Acute appendicitis	Cough with hemoptysis, weight loss
Lesion Location	Right anterior mediastinum	Left anterior mediastinum	Anterior mediastinum	Right anterior mediastinum	Left anterior mediastinum	Anterior mediastinum	Anterior mediastinum	Anterior mediastinum	Anterior mediastinum	Anterior mediastinum	Right anterior mediastinum	Right anterior mediastinum	Right anterior mediastinum	Left anterior mediastinum
Size（cm	12×12×10	10×10×15	N	16×12×12	9×5×6	12×6×9	13×8×10	15×12×16	8×6×12	8×8×11	8×9×11	7×9×10	9×8×10	12×8×9

3)														
Lesion Morphology	Round	Round	Round	Round	Lobulated	Lobulated	Lobulated	Lobulated	Lobulated	Lobulated	Round	Round	Round	Lobulated
calcification	N	N	N	punctate calcification	N	Little punctate calcification	Little punctate calcification	Little punctate calcification	-	-	-	-	-	-
cystic degeneration and necrosis	+	N	N	N	N	+	-	+	-	+	+	-	+	+
Enhancement Pattern	Inhomogeneous	N	N	N	N	Inhomogeneous	Inhomogeneous	Inhomogeneous	Inhomogeneous	Inhomogeneous	Inhomogeneous	Inhomogeneous	Inhomogeneous	Inhomogeneous

Pleural and Pericardial Effusion	Left pleural effusion	-	Bilateral pleural effusion	-	-	-	Bilateral pleural effusion	Right pleural effusion	-	Left pleural effusion	-	-	-	Left pleural effusion
Lymph Node Metastasis	Multiple enlarged lymph nodes in the upper mediastinum	-	-	-	Lymph node	-	Axillary lymph node metastasis	Supraclavicular lymph node	Lymph node	-	-	-	-	Mediastinal and hilar lymph nodes
Pulmonary Metastasis	Left Lower Lobe	-	-	Right Lung	Left Upper Lobe	-	-	-	-	-	-	-	-	Bilateral Lungs
Bone Metastas	Sternum	-	-	-	-	-	-	-	Multiple Bo	Multiple Bo	-	-	-	-

tasis									ne Metastases	ne Metastases				
Stage	Ⅳb	Ⅲ	Ⅳa	Ⅳa	Ⅲ	Ⅲ	Ⅳb	Ⅳb	Ⅳb	Ⅳb	Ⅲ	Ⅱ	Ⅱ	Ⅳb
Treatment	Chemotherapy + Radiotherapy	Resection + Radiotherapy+ Chemotherapy	Chemotherapy	Resection + Chemotherapy + Radiotherapy	Resection + Chemotherapy	Chemotherapy	Chemotherapy	Chemotherapy	Chemotherapy	Chemotherapy	Chemotherapy	Chemotherapy + Radiotherapy	Surgical Resection + Chemotherapy	Chemotherapy
Follow-up Outcome	15 months, dead	12 months, alive	11 months, dead	7 months, dead	29 months, alive	20 months, alive	12 months, dead	11 months, dead	1 month, alive	3 months, alive	19 months, alive, lost to follow-up	42 months,alive	13 months,alive	6 months,alive
